# Surgical and conservative management of medial plica syndrome: A systematic review and meta‐analysis of functional outcomes

**DOI:** 10.1002/jeo2.70473

**Published:** 2025-11-03

**Authors:** Piero Franco, Philipp Baumert, Fabrizio Di Maria, Angad Jolly, Fabrizio Matassi, Elisabeth Abermann, Riccardo D'Ambrosi, Christian Fink

**Affiliations:** ^1^ Department of Clinical Orthopaedics University of Florence Florence Italy; ^2^ Research Unit for Orthopaedic Sports Medicine and Injury Prevention Institute for Sports Medicine, Alpine Medicine and Health Tourism, FIFA Medical Centre of Excellence UMIT TIROL ‐ Private University For Health Sciences and Health Technology Hall in Tirol Austria; ^3^ School of Sport and Exercise Sciences Liverpool John Moores University Liverpool UK; ^4^ Department of General Surgery and Medical Surgical Specialties Section of Orthopaedics and Traumatology, University Hospital Policlinico “Rodolico ‐ San Marco”, University of Catania Catania Italy; ^5^ Department of Orthopaedics FMHS SGT University Gurugram India; ^6^ Gelenkpunkt Sports and Joint Surgery, FIFA Medical Centre of Excellence Innsbruck Austria; ^7^ IRCCS Istituto Ortopedico Galeazzi Milan Italy

**Keywords:** anterior knee pain, chronic pain, knee, plica syndrome

## Abstract

**Purpose:**

Knee plica syndrome is a common condition that often presents diagnostic challenges due to its symptom overlap with other knee pathologies. Surgical resection has traditionally been the common treatment; however, recent studies have questioned its necessity, proposing conservative interventions as effective alternatives. The aim of the current systematic review and meta‐analysis is to analyse and compare efficacy of surgical and conservative treatment.

**Methods:**

A comprehensive review and meta‐analysis was conducted on six clinical trials published between 2008 and 2023, focusing on surgical and conservative treatments for isolated knee plica syndrome. Rigorous inclusion criteria filtered studies that directly compared surgical and conservative methods. The risk of bias was assessed using the ROBINS‐I tool for nonrandomized studies and RoB‐2 for randomised trials. The studies were evaluated using patient‐reported outcome measures and the pain score. The meta‐analysis was carried out using the Lysholm Knee Score. Furthermore, complications and revisions surgery were recorded when reported.

**Results:**

The total number of patients investigated was 527 of which 284 were female (57%) and 209 males (43%). Patients were divided in two group according to the received treatment: both surgical and conservative treatments demonstrated improvements in knee function (*p* < 0.05). In the 12–24 month follow‐up period, the pooled mean difference for functional improvement was 24.5 (95% confidence interval [CI]: 18.7–30.2, *p* < 0.001), with no statistically significant difference favouring either approach (*p* > 0.05). However, heterogeneity was substantial (*I*² = 85.3%). No complications were reported with both treatment strategies.

**Conclusion:**

This study provides valuable insights into the treatment of knee plica syndrome, advocating for a conservative‐first approach. Establishing standardised protocols could enhance diagnostic precision and patient outcomes, ultimately optimising management strategies for this complex knee pathology.

**Level of Evidence:**

Level IV.

**Abbreviations**
CI: confidence intervals
EQ‐VAS: EuroQol‐visual analogue scales
KOOS: Knee Injury and Osteoarthritis Outcome Score
MINORS: Methodological Index for Nonrandomized Studies
MPP: medial patellar plica
MRI: magnetic resonance imaging
MVIC: maximal voluntary isometric contraction exercises
NHP: Nottingham Health Profile
NRS: numeric rating (pain) scale
PRISMA: Preferred Reporting Items for Systematic Reviews and Meta‐analyses
QoL: quality of life
RCS: retrospective case series
RCT: randomized controlled trial
REML: restricted maximum likelihood
ROBINS‐I: Risk Of Bias In Non‐randomised Studies ‐ of Intervention
RoB‐2: Risk of Bias‐2 (score)
RTS: return to sport
SD: standard deviation
VAS: Visual Analogue Scale
WMD: weighted mean differences

## INTRODUCTION

Navigating the complex landscape of knee pathologies, the diagnosis of plica presents particularly intricate challenge within orthopaedic field, and it is frequently underrecognized in clinical practice [16]. The synovial plica, a fold in the synovial membrane of the knee joint, can become a source of discomfort, leading to symptoms such as pain, clicking, or catching sensations [15, 23]. However, the uncertainty surrounding its diagnosis often complicates the treatment approach, as the symptoms of plica syndrome frequently overlap with those of other knee pathologies [2, 9, 15].

A central challenge in managing plica syndrome lies in the ambiguity surrounding optimal therapeutic strategies. Traditionally, surgical intervention has been a common recourse, which to date is performed arthroscopically, although the indications for the surgical resection are poorly defined. Recent investigations have increasingly highlighted the potential efficacy of conservative management approaches, which are demonstrating favourable outcomes in select patient populations [6, 20, 22].

Furthermore, methodological limitations in the existing literature make clinical decision‐making even more difficult, often failing to differentiate comorbidities present in the knee alongside plica syndrome, with the risk of inadequately reflecting the patient′s experience and the clinical benefit of surgical resection of the plica [2, 7, 9, 19, 23]. Moreover, a critical gap exists in the literature concerning the short‐term complications that may arise post‐surgery, such as hemarthrosis which could lead to variable pain, leaving a notable void in understanding the immediate aftermath of surgical interventions.

This systematic review and meta‐analysis aim to compare the efficacy and safety of conservative versus surgical treatment for isolated knee plica syndrome. By analysing clinical outcome scores and reporting on surgical complication this study seeks to clarify the therapeutic value of each intervention and inform future treatment guidelines emphasising the importance of precision in diagnosis and tailored treatment strategies for optimal patient outcomes.

## MATERIALS AND METHODS

### Literature search and selection criteria

To address this research purpose, a systematic review and meta‐analysis was designed and implemented. The study sample was composed of all articles on the treatment of the plica syndrome, enroling both surgical and conservative, published between 2008 and October 2023.

Inclusion criteria were as follows: full‐length articles and reports limited to clinical trials and cohort studies on the treatment of plica syndrome in the knee, including longitudinal studies (retrospective and prospective) and randomised controlled trials. The review did not restrict the type of surgical treatment or conservative approaches, including combined physical therapies and physiotherapy. There were no restrictions on the minimum number and age of people recruited. The minimum follow‐up period was 3 months.

Exclusion criteria were: case reports, expert opinions, previous systematic reviews, letters to the editor were excluded. Language was limited to English.

It was essential for the recruitment of the study that it had its own population and that there were outcomes measured by functional scores to allow objective comparability Reviews were examined for potentially relevant results method of comparing results. A computerised database search was performed using the PubMed, Cochrane Library, Embase, and Scopus databases. Specific ascertainment criteria were applied for the inclusion and exclusion of eligible studies. Two reviewers independently assessed study eligibility based on titles and abstracts. When eligibility could not be determined, full texts were reviewed. Disagreements were resolved through discussion, and if consensus could not be reached, the senior Author was consulted to make a final decision.

The search strategy was initially developed for PubMed and then adapted for each subsequent database. The search terms included: (“plica”[All Fields] OR “plicae”[All Fields] OR “plicas”[All Fields]) AND (“knee”[MeSH Terms] OR “knee”[All Fields] OR “knee joint”[MeSH Terms] OR (“knee”[All Fields] AND “joint”[All Fields]) OR “knee joint”[All Fields]). Specifically, the terms 'plica' and 'knee' were combined using the 'Title, Abstract, Keywords' search function in the Embase, Scopus, and Cochrane databases. The authors applied filters to include articles published between 2008 and 2023.

### Assessment of the risk of bias in included studies

The assessment of risk of bias in the included studies was undertaken independently and in duplicate as part of the data extraction process and in accordance with the approach based on the Centre for Evidence‐Based Medicine, Critical Appraisal of Prognostic Studies work‐ sheet. The ROBINS‐I tool was used for no randomised studies and RoB‐2 was used for the randomised clinical trials included.

Once the studies were selected, the quality of the selected studies was evaluated using the Methodological Index for Nonrandomized Studies (MINORS) score [25]. The checklist includes 12 items, of which the last four are specific to comparative studies. The minimum score accepted was ≥12 for the retrospective or prospective studies, and ≥20 for the randomised control trial [25]. Key inclusion criteria required studies to focus on a defined population without knee joint comorbidities, except for associated cartilage lesions, and to report outcomes using a clinical scoring system, enabling meta‐analytic comparability and meaningful data interpretation.

### Data collection and analysis

Data were extracted independently by two reviewers. The data collected from the studies included the number of patients treated, age, sex, BMI and patient reported outcomes measured and pain score. Moreover, any comorbities of the knee, such as cartilage damage was reported, if presented and consecutively classified by the entity of the damage. Any possible perioperative complication after surgery was reported, along with possible revision surgery.

Different methods of diagnosis for the medial plica syndrome were noted in different studies.

The population was divided into two groups: a surgical treatment group (Group S) and a conservative treatment group (Group C). In studies that included both surgical and conservative treatment arms, patients were analysed as two separate subgroups within the same study. This allowed direct comparison between treatment modalities while preserving the internal structure and integrity of each original study for the purposes of meta‐analysis.

### Statistical analysis

The meta‐analysis was conducted using R (Datacamp Inc. New York, USA), a statistical computing environment using the packages meta for meta‐analysis [24], and ggplot2 [27] for visualisation, and other packages for reproducible research (including dplyr and gridExtra). For the meta‐analysis, weighted mean differences (WMD) with 95% confidence intervals (CI) and standard errors were calculated for each study. Heterogeneity was assessed using the I² statistic, with thresholds for low ( < 25%), moderate (25%–75%), and high ( > 75%) heterogeneity, and τ² (tau‐squared) to estimate between‐study variance. A random‐effects model was applied using the restricted maximum likelihood (REML) approach when τ² > 0 and *I*² exceeded 25%, indicating moderate or greater heterogeneity. Missing standard deviations (SDs) were imputed using a weighted mean SD derived from available data, ensuring consistent representation of study variability. The meta‐analysis employed a random‐effects model, incorporating subgroups based on intervention type (surgical vs. conservative). To evaluate robustness, a sensitivity analysis was conducted by recalculating the meta‐analysis, sequentially omitting each study. Finally, a forest plot was generated to illustrate WMDs across subgroups.

## RESULTS

### Search results

**Figure 1:**
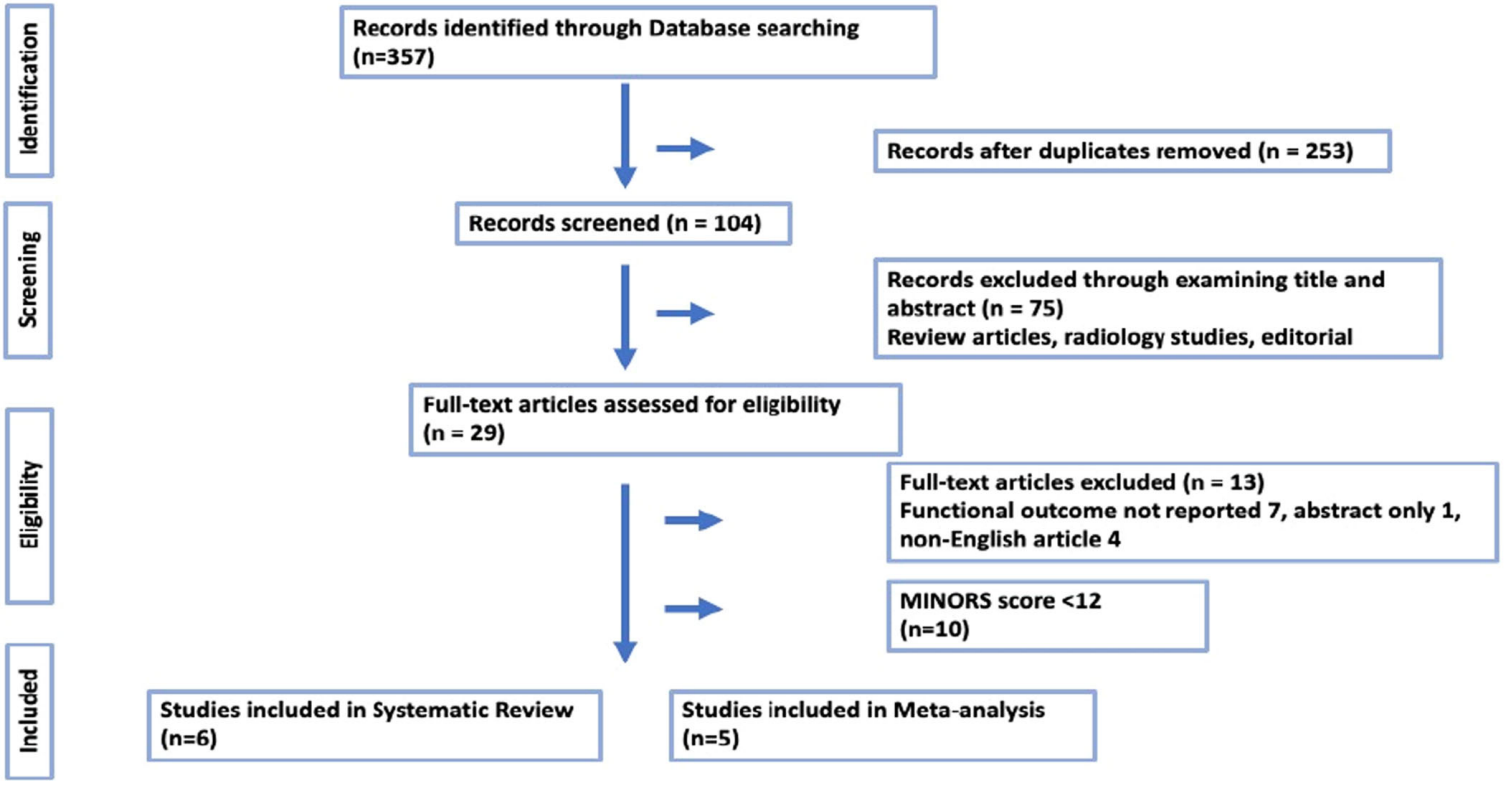
Article selection flowchart.

An initial search identified 357 records. After removal of duplicates, 104 unique abstracts were screened, leading to 29 studies selected for full‐text review. Three articles were not written in English, and the remaining studies were evaluated using the MINORS score, which is highlighted in Table 1, ultimately resulting in the inclusion of six studies. Ten studies were excluded from the systematic review due to a MINORS score below the pre‐set threshold. However, Hufeland et al. [11]. was later excluded from the meta‐analysis due to the lack of a common outcome measure for functional outcomes and pain score with the other studies. Figure 1 shows the flowchart of studies selection process, according to PRISMA guidelines.

**Table 1:** Methodological Index for Non‐Randomised Studies (MINORS) score detailed subheading assessments for each included study in the systematic review.

Author′s name	Clear stated aim	Inclusion of consecutive patients	Prospective data collection	Appropriate endpoints to the aim of study	Unbiased assesment of the study	Appropriate perioperative follow‐up	Loss of follow‐up less then 5%	Prospective calculation of study size	Adequate group control	Contemporary group	Baseline equivalence of group	Adequate statistical analyses	Total score
Blanke et al. [2]	2	2	2	1	1	2	2	2	N/A	N/A	N/A	N/A	15
Hufeland et al. [11]	2	2	2	2	1	2	2	1	N/A	N/A	N/A	N/A	14
Prejbeanu et al. [20]	1	2	1	2	2	2	0	2	N/A	N/A	N/A	N/A	12
Paczesny et al. [19]	2	2	2	1	1	2	1	1	N/A	N/A	N/A	N/A	12
Sauer et al. [22]	2	2	2	2	2	2	2	2	2	2	2	2	24
Genc and Duymaz [6]	2	2	2	2	2	1	2	1	2	2	2	1	21

*Note*: The last four items (adequate group control, contemporary group, baseline of equivalence of group and adequate statistical analyses) are specifically reserved to RCT (randomised control trial).

The meta‐analysis was carried out using the Lysholm score, since it was the only reported in more than two studies. The statistical analysis was divided in two end points: at the short term (3–6 months) the Lysholm score was reported in three studies, and at long‐term (12–24 months) in four studies.

### Study design and characteristics

**Figure 2:**
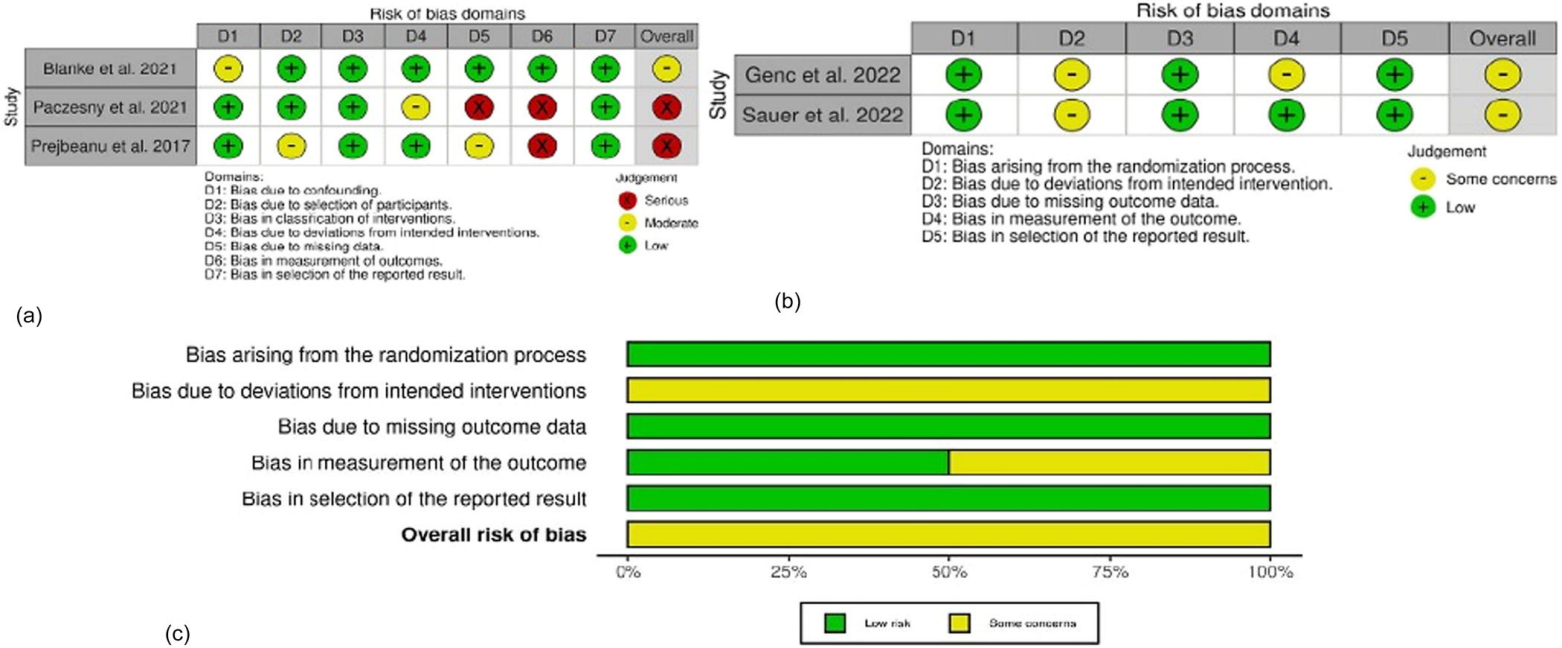
BIAS assessment; (a) ROBINS score for retrospective and prospective studies, some concern regards missing data and outcome measure. (b) RoB score for RCTs some concern for bias due to deviations from intended interventions and bias from outcome measurements. (c) Overall bias measures, there are some concerns regarding overall risk of bias, final results can be taken for granted; there is no high level of evidence. RCT, randomised control trial; RoB‐2, Risk of Bias‐2 (score); ROBINS‐I, Risk Of Bias In Non‐randomised Studies ‐ of Intervention.

The population characteristics of each study are shown in Table 2. The total population is 527 of 312 female (57%) and 219 males (43%). The surgery group was composed of 231 female (60%) and 155 male (40%) for a total of 382 patients (72%), the conservative group was composed of 145 (28%) patients, which were divided 81 female (56%) and 64 male (44%). The mean age of participants in the surgical group was 23.4 ± 5.9, and in the conservative group 31.8 ± 6.1 years (*p* < 0.001). Among the six included studies for the systematic review: two were a retrospective study, two prospective cohort study and two randomised control trial. The risk of bias of selected studies was determined using ROBINS and RoB for the retrospective studies and the two randomised studies, respectively. Figure 2 shows the precise score for each feature of RoB and ROBINS. The only type of plica reported in the studies was the medial plica, expect for Hufeland et al. [11], who has reported two cases of infrapatellar and suprapatellar plica. If there were intraoperative findings of supra‐ or infrapatellar plica, no surgical management was undertaken.

**Table 2:** Population features.

	Author	Study design	Type of plica	Plica classification	Plica class. distribution	N. of patients	Follow‐up median (m)	Time onset of pain‐ (mean, month)	Age of patients (mean)	Female/Male	BMI	Knee comorbidities	Type of Comorbidities	Type of Lesion	Type of Diagnosis:	Pain evaluation	Type of score/outcome	RTS
GROUP S																		
	Blanke et al. [2]	RCS	Medial	Not available; diameter of plica e area of contact with cartilage	N/A; surgical treatment significant larger diameter	76	12	2	28	44/32	N/A; <30	No; exclusion criteria	N/A	N/A	MRI + MPP TEST	VAS	Lysholm, Tegner	YES
	Prejbeanu et al. [20]	RCS	Medial	Sakakibara	A 3 (1.5%) B 28 (14%) C 105 (52%) D 64 (32%)	200	36	12	28	108/92	N/A	Yes	Chondral Lesion medial condyle	Outerbridge 0‐28 (14%) 1‐120 (60%) 2‐41 (20,5%) 3‐11 (5.5%)	MRI	EQ‐VAS	Lyshom	N/A
	Hufeland et al. [11]	PCS	Medial	Sakabira	B 10 (26%) C 28 (73%); infrapatellar 1 suprapaterllar 1 (not treated	34	20	27	16	28/10	N/A	No Exclusion criteria	N/A	N/A	MRI	NRS	Koos, Kujala, Tegner	N/A
	Paczesny et al. [19]	PCS	Medial	Sakakibara	A (7%)B (31%) C (60%) D (2%)	48	36	26	18	32/12	N/A	Yes	Chondral Lesion	ICRS 0 = 39; I = 3; II = 6; III = 4	MPP + US	N/A	Lyshom, Tegner; MVIC, QoL	N/A
	Sauer et al. [22]	RCT	Medial	Sakakibara	Not specified the number (A and B)	24	24	2	27	19/5	N/A	N/A	N/A	N/A	MRI + MPP TEST	VAS	Lyshom, Kujala, QoL	N/A
GROUP C																		
	Blanke et al. [2]	RCS	Medial	Not available; diameter of plica e area of contact with cartilage	N/A; surgical treatment significant larger diameter	41	12	2	26	22/19	N/A; <30	No; exclusion criteria	N/A	N/A	MRI + MPP TEST	VAS	Lysholm, Tegner	YES
	Sauer et al. [22]	RCT	Medial	Sakabira	Not specified the number (A and B)	24	24	2	27	14/10	N/A	N/A	N/A	N/A	MRI + MPP TEST	VAS	Lyshom, Kujala, QoL	N/A
	Genc and Duymaz [6]	RCT	Medial	N/A	N/A	80	7	15	37	45/35	27	No; exclusion criteria	N/A	N/A	MRI; MPP test	VAS	Lyshom; tuds tes for strenght; QoL NHP	N/A

*Note*: Group C, conservative treatment group; Group S, surgical treatment group.

Abbreviations: EQ‐VAS, EuroQol‐visual analogue scales; KOOS, Knee Injury and Osteoarthritis Outcome Score; MPP, medialpatellar plica (test); MRI, magnetic resonance imaging; MVIC, maximal voluntary isometric contraction exercises; NHP, Nottingham Health Profile; NRS, numeric rating (pain) scale; QoL, quality of life; RCS, retrospective case series; RCT, randomised controlled trial; RTS, return to sport; VAS, Visual Analogue scale.

The diagnostic protocol for such a rare condition was a combination of clinical and instrumental examination, which could vary between studies. In the selected studies, the most common clinical examination was the medio‐patellar plica (MPP) test [2, 6, 19], which could be combined with the presence of chronic anteromedial knee pain, which was itself suggestive of patellar plica syndrome [2, 11, 20] However, the clinical examination alone was often insufficient for arriving at a diagnosis, with the consensus being that instrumental examinations were necessary. These included MRI [2, 6, 20] and, alternatively, the dynamic ultrasound technique described in Paczesny′s study [19]. However, Sauer did not specify how medial plica was assessed [22]

The surgical treatment was exclusively performed using an arthroscopic approach, with no cases involving arthrotomy. Procedures addressing associated cartilage damage were not specified, apart from the studies of Paczesny et al. and Prejbeanu et al., which was considered exclusion criteria [19, 20]. The method of resection was not consistently reported, and when mentioned, no standard technique could be established, as both radiofrequency and shaver methods were documented as options. Surgical treatment was never suggested as a first‐line treatment, but various therapies were tried before patients underwent arthroscopic resection. No specific protocol of preoperative conservative treatment was followed in different studies: in Blanke′s study, all patients tried at least 3 months of conservative treatment in different institutions [2]. Conservative treatment included activity modification, sports restriction, physiotherapy with range of motion exercises, physical applications (cryotherapy and electronic stimulation) and NSAIDs. In the RCT by Sauer et al., every patient had a mandatory 3 months of physiotherapy [22]. In the retrospective analysis by Prejbeanu et al., patients enroled for surgery had chronic knee pain that was not relieved by NSAIDs and had benefited from quadriceps exercises for at least 3 months prior to surgery [20]. Paczesny et al. enroled patients for surgical treatment after persistent symptoms despite 6 months of physiotherapy, which was discontinued at another centre [19]. No complications associated with surgery, such as post‐operative swelling, duration of any lameness and use of crutches, have been reported in any case. Only Blanke et al. [2] reported the use of post‐operative drainage, though the timing of its removal was not specified. Additionally, it was noted that the patient used crutches for at least one week. All patients continued with a course of physiotherapy following surgery, with a focus on an immediate recovery of full range of motion in the joint, and several authors emphasise the importance of massage and passive mobilisation of the patella, i.e. there is a focus on avoiding surgical scarring. There is no evidence of arthrofibrosis and post‐surgical joint stiffness or timing of full ROM recovery.

A conservative treatment protocol was reported in three studies, each with varying details but sharing a common underlying approach. No episodes of intra‐articular infiltration of drugs were reported after surgery or during conservative treatment.

The duration and frequency of conservative treatment varies in the three studies in the conservative group. Blanke et al. [2]. reports sessions two to three times a week for 3 weeks accompanied by the physiotherapist. Sauer et al. [22] reports a 3‐month physiotherapy cycle in which, however, the physiotherapist′s sessions were only once a week for the first month to better explain exercises. Finally, Genc and Duymaz [6] reports that one physiotherapist performed the exercises 5 days per week over 6 weeks in the physical therapy clinic on a short schedule, the patients were then led to perform the exercise scheme on their own and were followed up by telephone for updates twice a week.

The major focus of the physiotherapy cycle in all three studies is quadriceps muscle strengthening, combined with stretching. Sauer et al. [22], in particular, specifies that he avoided open kinetic chain exercises with overloads as a potential source of inflammation for the plica, with the exception of straight‐leg lifts.

### Patient‑reported outcome measures and return to sports activity

The protocols varied among the recruited studies. The Lysholm knee score was reported in 489 (92% out of 527 of the total population recruited in the systematic review) patients from five studies. Return to sport has only been investigated by Blanke et al. [2] and only at a long‐term time point: 1 year after surgery. Blanke et al. assessed the percentage of the population that returned to sport following surgical treatment, reporting a rate of 97%. The Tegner score was reported in two studies, so it was not included in the meta‐analysis. Additional outcome measures were reported by Sauer et al. and Hufeland et al. [11, 22], who utilised the Kujala score, also known as AKPS (anterior knee pain score) and by Genc and Duymaz [6], who employed scores on specific functional strength tests and different scales of quality‐of‐life measurement.

Pre‐ and postoperative perceived pain was collected in a disparate manner, different measurement scales being reported, as can be seen in Table 3, and therefore not included in a meta‐analysis. The most commonly used scale was the VAS used by Blanke et al. and Sauer et al. [2, 20]. While Hufeland et al. [11] used the NRS scale. In general, all the studies show a downward trend compared to the pre‐operative values that is also maintained over the long term, this can be observed in both the S and C groups. Patient′s reported outcome and VAS are specifically reported in Table 3.

**Table 3:** Functional Outcomes.

	Author	Pre‐treatment VAS scale	Post‐treatment VAS scale short term (6 m)	Post‐treatment VAS scale long term (after 12 m)	Pre treatment‐Lysholm score	Post treatment‐Lysholm score short term (6 months)	Post treatment‐Lysholm score long term (12 months)	Pre treatment‐Tegner score	Post treatment‐ Tegner score short term (6 months)	Post treatment‐ Tegner score long term (12 months)	Pre treatment‐Kujala score	Post treatment‐ Kujala score short term (6 months)	Post treatment‐ Kujala score long term (12 months)
GROUP S													
	Blanke et al. [2]	N/A	N/A	N/A	64	N/A	95.9	3.6	N/A	6	N/A	N/A	N/A
	Prejbeanu et al. [20]	N/A	N/A	N/A	68.2	90.8	94.7	N/A	N/A	N/A	N/A	N/A	N/A
	Hufeland et al. [11]	N/A	N/A	N/A	N/A	N/A	N/A	2.2	N/A	4.9	52	N/A	80
	Paczesny et al. [19]	N/A	N/A	N/A	52	75.3	78.7	N/A	N/A	N/A	N/A	N/A	N/A
	Sauer et al. [22]	7	3.3	2.3	65.8	81.5	89	N/A	N/A	N/A	69.4	83.95	90.2
GROUP C													
	Genc and Duymaz [6]	7	5	N/A	45.1	70	N/A	N/A	N/A	N/A	N/A	N/A	N/A
	Blanke et al. [2]	N/A	N/A	N/A	70	N/A	96	3.9	N/A	6	N/A	N/A	N/A
	Sauer et al. [22]	7	4.5	4.7	66.3	79	72.6	N/A	N/A	N/A	70.6	76.8	75

*Note*: Group C, conservative treatment group. Group S, surgical treatment group.

Abbreviation: VAS, Visual Analogue Scale.

### Meta‐analysis of Lysholm score

Commonly reported outcome measures include only the Lysholm Knee Scoring Scale, which were consistently used in the recruited studies. The meta‐analysis was therefore conducted on the Lysholm score only.

Regarding the Lysholm score, the total population was divided into two groups: A conservative treatment and a surgical treatment group. The randomised controlled trial by Sauer et al. [22] included both surgical and conservative treatments. Three studies were included in short‐ (3–6 months) and four studies in long‐term (12–24 months) follow‐up analyses, respectively. The total number of patients for the surgery group was 382 (231 females/151 males) The conservative group was composed of 145 (81 females/64 males). At short term, the recruited patients were 264 in the surgery group and 97 in the conservative group. At the long term the patients were 295 in the surgery group and 138 in the conservative group.

**Figure 3:**
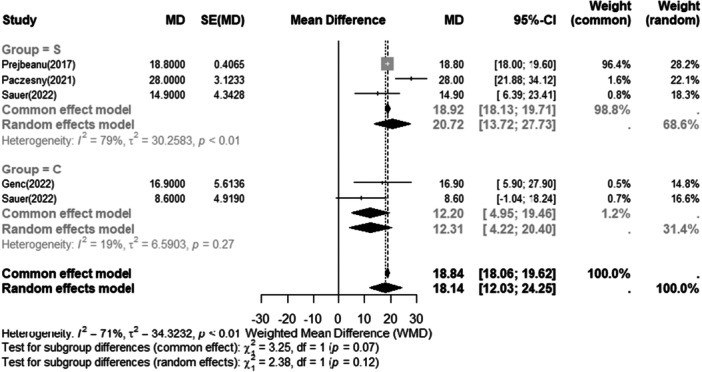
Meta‐analysis of short‐term results (3–6 months): forest plots showing Lysholm score between conservative (−) and surgery (+). The weighted mean differences (WMD) and standard error (SE) are reported for continuous and categorical variables, respectively. A 95% confidence interval (CI) is reported.

**Figure 4:**
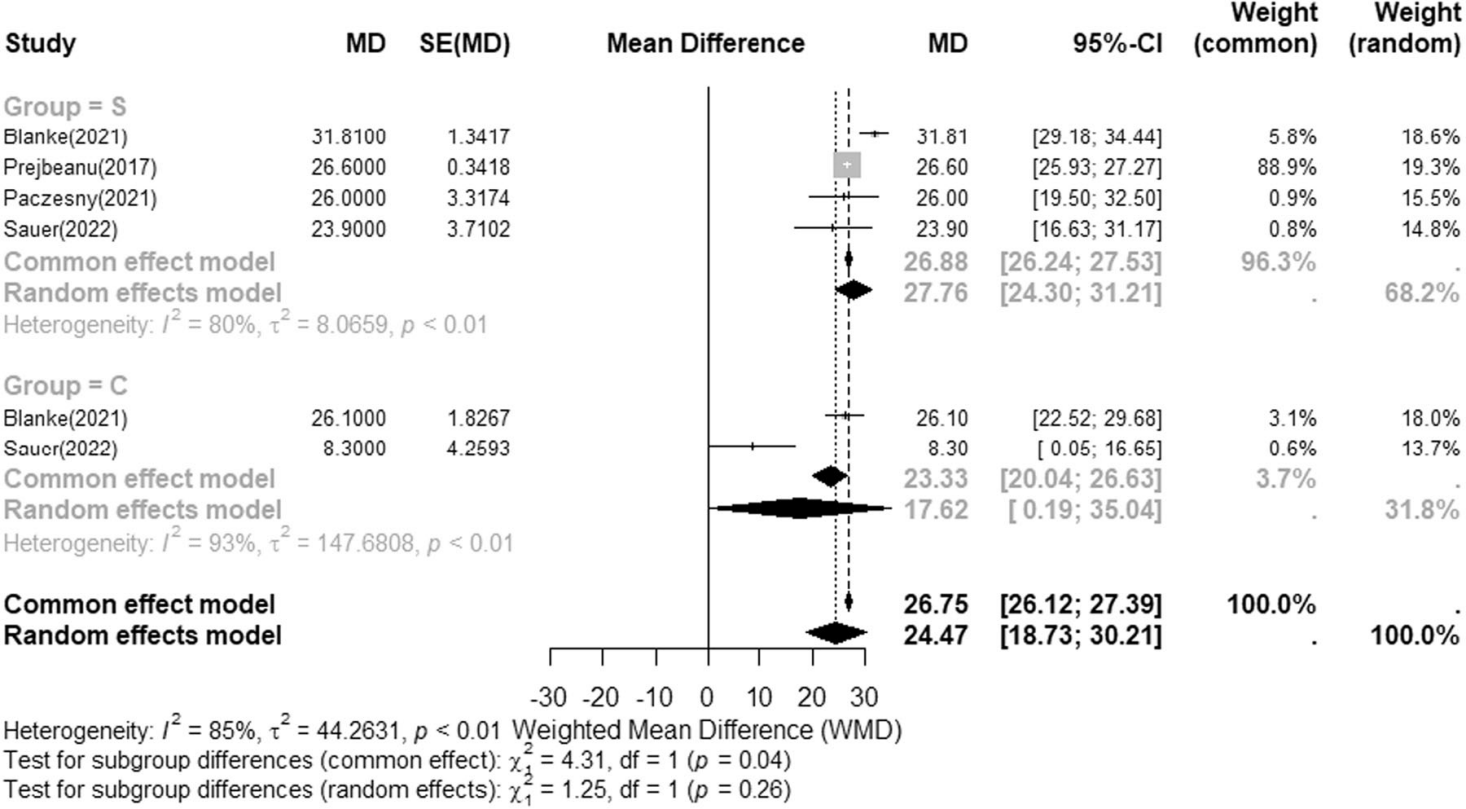
Meta‐analysis of long‐term results (12‐24 months): forest plots showing Lysholm score between conservative (−) and surgery (+). The weighted mean differences (WMD) and standard error (SE) are reported for continuous and categorical variables, respectively. A 95% confidence interval (CI) is reported.

The short‐term and long‐term Lysholm score results are listed in Figures 3 and 4, respectively.

Cochran′s Q‐test indicated substantial heterogeneity in both the short‐term (Q = 13.89, *p* < 0.001) and long‐term (Q = 33.95, *p* < 0.001) follow up analyses, which may be attributable to clinical heterogeneity and limited specification of inclusion criteria across the included studies. The forest plots Figures 3 and 4 show the delta of improvement of Lysholm from preoperative to postoperative. The pre‐operative and post‐operative Lysholm scores for each study are presented in Table 3. Both interventions were associated with overall improvement, any difference in the outcome between the two treatments did not reach statistical significance (*p* > 0.05).

A sensitivity analysis was conducted, where one study at a time was removed from the meta‐analysis. This revealed no substantial differences in the overall outcomes for both short‐ and long‐term follow‐up. Regardless of these adjustments, the Lysholm score consistently improved, with no significant differences between the treatment modalities.

## DISCUSSION

The purpose of this meta‐analysis was to determine the relative effectiveness of conservative versus surgical management in treating isolated plica syndrome. The main finding in this meta‐analysis reveals that both treatments improve the condition without significant differences between the treatments. Regarding the Lyshom score, conservative treatments over the three studies seems to give positive results, which are stable in the long term. Even if the data regarding the pain score were fragmentary, the pain score seems to have the same trend [22]. Therefore, the meta‐analysis does not suggest any surgical treatment over conservative treatment for the plica syndrome. The interpretation stems from two elements. Firstly, the meta‐analysis demonstrated that, over time, conservative treatment yielded comparable results to surgery for patients with similar demographic features and population. Secondly, although surgery was proposed as a second‐line treatment following unsuccessful conservative treatment, the protocol that patients had followed was not specified. It is evident from the Sauer RCT [22] that the random assignment of surgical or conservative treatment after three months of physiotherapy does not result in statistically significant differences in functional scores between the two groups. This suggests that patients' adherence to physical therapy protocols in the long term is more crucial for achieving clinical outcomes. However, the presented study fails to address any common pre‐operative clinical signs that could indicate which patients may benefit from surgery after brief conservative treatment.

Greater attention must be given to conservative treatments for plica syndrome. It is challenging to estimate the real effect of plica excision, especially when associated with other pathologies. The potential benefits of surgery are unclear. Strengthening the quadriceps and improving patellar tracking through conservative measures, such as physiotherapy, appears promising given the plica′s connection to the quadriceps tendon [7, 15]. Nevertheless, surgical resection of the plica might be considered after a thorough conservative rehabilitation protocol and exclusion of other pain causes, which was already highlighted from previous research [1, 3, 8, 16, 26].

It is curious that there was no description regarding short term post operative complication in the Group S such as hemarthrosis or knee pain following surgery. This omission is particularly unusual, given that synovial resection is expected to cause bleeding in the joint. Such information would be highly valuable when deciding whether to resect the plica if it is incidentally identified during arthroscopy. Moreover, no reported complication is a contrast data with a recent survey carried out in 2024 among the German Knee Society, in which 200 surgeons reported overall 11% rate of complication after medial plica resection, interesting data arise from the high volume arthroscopy surgeon, who reported a way higher complication rate, up to 50%. Hemarthrosis and persistent pain were the more common events [5].

The differential diagnosis of plica syndrome and its contribution to anterior knee pain remains a significant challenge. Isolated studies focusing solely on plica pathology are scarce, complicating the understanding of this condition. Furthermore, preoperative classification of plica morphology shows no clear correlation with the necessity for surgical intervention, as highlighted in current literature [2, 15]. This underscores the difficulty in using morphology as a standalone criterion for deciding on surgery.

Moreover, the majority of the analysed studies have included several patients with concomitant cartilage lesion, with Prejbeanu et al. [18] being an exception. While it is known that a medial plica can cause cartilage damage, the articles do not specify the location of the damage or detail the surgical procedures performed. This lack of information has been identified by the authors as a significant bias when evaluating surgical outcomes [2, 3, 4, 11, 19, 20, 22, 26].

It remains unclear when the plica becomes a source of pain or contributes to cartilage damage in the trochlea, as clinical examinations alone cannot reliably determine this. Blanke et al. [2] suggests a correlation between the size of the plica on MRI and the need for intervention. The aforementioned study offers an innovative perspective compared to other studies. However, it has reproducibility limitations as the proposed treatment cut‐off is below 1,5 millimetres and requires a high magnetic field MRI. Such technology is not always available in all settings [2, 12, 15]. Moreover, the plica being a remnant of synovial tissue remains reactive to inflammation so the size of the plica itself may vary and thus the surgical indication may be strongly influenced with respect to the timing of the MRI [17].

The optimal treatment for plica syndrome is not well‐defined. Current literature suggests proposing surgical treatment after failed physiotherapy. At date the conservative treatment options are under‐investigated compared to surgical interventions [8, 23]. There is also no standardised physiotherapy protocol for plica syndrome, and there are no clear guidelines on the necessity of physiotherapy post‐surgery. Techniques such as Kinesio taping have shown potential, but more rigorous studies are needed to validate their efficacy [6].

Medial plica can be classified by Sakakibira arthroscopic classification [21], which appear to be the most commonly used classification across studies. However, this classification is often refined during arthroscopy rather than preoperative evaluation, limiting its utility in determining surgical indications. This creates a challenge in assessing, on an outpatient basis, the contribution of the medial plica to anterior knee pain based solely on MRI findings.

Significant heterogeneity was observed in both the outcome measures and their timing, making it challenging to interpret medial plica syndrome consistently and complicates the ability to conduct a high‐quality meta‐analysis. In particular, the lack of data reported in the studies by Paczesny et al. and Prejbeneau et al. increases the risk of bias [19, 20].

In addition, few studies were selected that did not include other confounding pathologies of the knee, as reflected in the MINORS score. This limits the ability to generalise the results specifically to the plica syndrome: it has already been pointed out by Paczesny et al. that cartilage damage classified with ICRS above Grade 2 has an important influence on functional scores. This is an expected outcome, and one might wonder whether it is therefore not the cartilage lesion per se that limits the outcome, and any corrective surgical procedures for the cartilage might not have more relevance than resection of the plica. Notably this aspect was not investigated by Paczesny et al. 145 (28%) patients or addressed in other studies [19].

The study of Paczesny et al. also brings to our attention a very interesting point of view in the clinical evaluation of these patients, namely how dynamic valgus could give worse results than plica resection, causing cartilage damage due to overloading of the patellofemoral joint, along with which a hypo functioning vastus medialis oblique can also affect the results. What Paczesny et al. finally suggests is that such patients with impaired patellar tracking should not be treated with plica resection alone, but that correction of the biomechanics should also be considered, although it′s not specified which biomechanical corrections are suggested.

This suggestion linking medial plica symptomatology to the patient′s biomechanical functional picture is supported by the randomised studies of Genc and Duymaz and Sauer et al. Both studies demonstrate that a structured functional rehabilitation protocol, with or without taping treatment, leads to improved results. In the study of Sauer et al., these results were comparable to surgical intervention, a finding which is also reconfirmed by this meta‐analysis [6, 22].

The Kujala score, focusing on anterior knee pain, may offer a more accurate assessment, as emphasised by Sauer et al., who stressed the importance of precise outcome measurement [12, 14]. Additionally, short‐term return to sport and surgical complications were inadequately investigated.

Tracking return to sport in the treatment of plica syndrome might be a further perspective worth exploring, especially given the young and typically active population observed in this study. Blanke et al. reported an almost complete return to sport within one‐year post‐surgery in the study population [2]. Measuring this variable at such a long time point, however, does not give a true clinical correlation to the extent of the pathology, as even major surgical procedures such as ligament reconstruction would predict a return to sport in less than one year [4, 10, 13].

Despite long term outcome has utmost importance to define the success of a procedure, plica resection should be studied more precisely in the short term after surgery, in particular hemarthrosis post resection and immediate post operative pain ought to be investigated, this would be of value in finding out whether the resection of the plica might be worthwhile if it is occasionally found during arthroscopy performed for other reasons.

What remains unclear, despite the extensive literature search, is when a medial plica actually becomes pathological, causing cartilage lesion and warranting treatment. This meta‐analysis suggests that, given the general trend of clinical and functional improvement with both surgical and conservative treatment, there appears be no clear indication for resection of the plica. Furthermore, the authors' opinion, supported by this meta‐analysis, is that plica resection during arthroscopy performed for other pathologies has no reason to be performed.

## LIMITATIONS

The results need to be cautiously interpreted due to the low number of recruited studies and high heterogeneity among them. This variability likely stems from differences in patient selection, some studies might have chosen different patient criteria, which could skew the outcomes. Comorbidities, some studies accounted for comorbid conditions,while others did not, leading to inconsistencies.

Furthermore, the lack of uniform exclusion criteria for conditions like cartilage damage contributed to heterogeneity. The statistical analysis is hampered by the quality of the selected studies, which generally scored below the optimal threshold for low risk of bias. The lack of uniformity in study methodology and outcome measures further complicates analysis. The meta‐analysis was limited to the Lysholm score. However, this score alone is insufficient to provide a comprehensive understanding of plica syndrome. The studies examined exhibit a moderate risk of bias according to the ROBINS‐I and ROB2 assessments; when combined with the low MINORS scores, this further diminishes their overall scientific validity.

## CONCLUSION

This research represents the first attempt to systematically compare different cohorts over both the short and long term. The meta‐analysis shows comparable improvements in Lyshom score and pain reduction after both surgical and conservative treatment. Despite its limitations, there is no evidence supporting the superiority of surgical procedures for the medial plica syndrome. Future research should focus on developing standardised diagnostic criteria, preoperative precise classification, exploring conservative treatment efficacy and establishing clear guidelines for surgical intervention and postoperative care. The analysis was limited by high heterogeneity across studies and the absence of standardised treatment protocols, impacting the ability to generalise findings.

## AUTHOR CONTRIBUTIONS

Christian Fink contributed to the study conception and design. Screening and data collection were performed by Piero Franco and Angad Jolly. Risk of bias assessment was conducted by Fabrizio Di Maria and. Data analyses were performed by Philipp Baumert. Piero Franco and Philipp Baumert assessed the interpretation of the data. Christian Fink supervised the study from inception. The first draft of the manuscript was written by Piero Franco. Riccardo D'Ambrosi, Elisabeth Abermann and Fabrizio Matassi reviewed the draft and all authors commented on previous versions of the manuscript. All authors read and approved the final manuscript.

## CONFLICT OF INTEREST STATEMENT

The authors declare no conflicts of interest.

## ETHICS STATEMENT

Prospero Registry Number CRD42023478277. All patients provided informed consent before enrolment in the study.

## Data Availability

The data sets used and/or analysed during the current study are available from the corresponding author on reasonable request.
